# Anti-ableism and scientific accuracy in autism research: a false dichotomy

**DOI:** 10.3389/fpsyt.2023.1244451

**Published:** 2023-09-08

**Authors:** Kristen Bottema-Beutel, Steven K. Kapp, Noah Sasson, Morton Ann Gernsbacher, Heini Natri, Monique Botha

**Affiliations:** ^1^Lynch School of Education and Human Development, Boston College, Boston, MA, United States; ^2^Department of Psychology, University of Portsmouth, Portsmouth, United Kingdom; ^3^School of Behavioral and Brain Sciences, University of Texas at Dallas, Dallas, TX, United States; ^4^Department of Psychology, University of Wisconsin–Madison, Madison, WI, United States; ^5^The Translational Genomics Research Institute, Phoenix, AZ, United States; ^6^Psychology Department, Stirling University, Stirling, United Kingdom

**Keywords:** autism, stigma, ableism, bias, anti-ableism

## Abstract

It was recently argued that autism researchers committed to rejecting ableist frameworks in their research may sacrifice “scientifically accurate” conceptualizations of autism. In this perspective piece, we argue that: (a) anti-ableism vs. scientific accuracy is a false dichotomy, (b) there is no ideology-free science that has claim to scientific accuracy, and (c) autism science has a history of false leads in part because of unexamined ableist ideologies that undergird researcher framings and interpretations of evidence. To illustrate our claims, we discuss several avenues of autism research that were promoted as scientific advances, but were eventually debunked or shown to have much less explanatory value than initially proposed. These research programs have involved claims about autism etiology, the nature of autism and autistic characteristics, and autism intervention. Common to these false leads have been ableist assumptions about autism that inform researcher perspectives. Negative impacts of this work have been mitigated in some areas of autism research, but these perspectives continue to exert influence on the lives of autistic people, including the availability of services, discourses about autism, and sociocultural conceptualizations of autistic people. Examining these false leads may help current researchers better understand how ableism may negatively influence their areas of inquiry. We close with a positive argument that promoting anti-ableism can be done in tandem with increasing scientific accuracy.

## Introduction

### Ableism and anti-ableism in autism research

Autism research has been criticized for being *ableist* (1–3). Ableism refers to a system of discrimination against people perceived to be disabled, based on socially constructed views of “normalcy, productivity, desirability, intelligence, excellence, and fitness” (4). A feature of this system of discrimination for autistic people is stigmatization, which can mean that autistic characteristics such as developing passionate interests on topics that others consider unusual or otherwise not adhering to social norms, are devalued in both interpersonal interactions and broader social contexts (5). Stigma is associated with less knowledge of autism, greater interest in curing and normalizing autistic people, and less familiarity with autistic people [e.g., not having an autistic family member; (6)]. Stigmatization can have significant negative impacts on autistic people’s lives, such as lowered life expectancies, under-employment, and lowered quality of life (7).

Autism research focuses almost exclusively on autistic people’s perceived deficits relative to non-autistic people, and researchers rarely acknowledge that autistic people have strengths and abilities in addition to impairments, and exist in contexts that enable or disable functioning. Autistic people are often inaccurately described as missing core human capacities (8), and as incapable of social reciprocity or contributing to shared culture (2). Deficit construals persist even when autistic people show strengths in domains that would otherwise be considered positive, such as transparency, rationality, and morality (9–11), and this framing is encouraged in common nosologies (e.g., the Diagnostic and Statistical Manual of Mental Disorders and the International Classification of Disabilities). Illustrating this issue, in a study investigating how almost 200 autism researchers construct autistic people, two-thirds of accounts included at least one dehumanizing, objectifying, or stigmatizing statement (2). Persistent negative evaluations of autistic people in the face of contrary evidence point to deeply ingrained social and cultural values about autism that influence researchers’ interpretations of their findings.

There has been growing attention to how ableism in autism research impacts the scope and quality of research available (1–3, 11, 12). Exclusively focusing on deficits does not represent autistic people 12; or autism accurately; instead it reflects the interests of primarily non-autistic researchers. In contrast, autistic accounts of autism tend to be broad in scope, rather than deficit-focused (13), and co-produced work tends to advocate for a holistic approach to understanding autistic strengths and challenges (14, 15). Additionally, including autistic people in autism research is associated with lower odds of having ableist constructions of autism or autistic people (2, 16).

### Backlash: rejecting anti-ableism in the guise of scientific accuracy

Many autism researchers have embraced calls to dismantle ableism in their work (17). However, others have asserted these efforts hamper scientific accuracy, particularly in regards to discourses and terminology used to describe autistic people (18). Their argument is that some autistic people– especially those who have accompanying intellectual disability, do not consistently use speech, and/or require substantial support– cannot be described without using terms such as “profound,” “severe,” and “problem behavior,” which many autistic people and their allies find dehumanizing (3, 19). These terms are proposed to be fact-based, scientifically accurate descriptors that, if abandoned, would leave researchers unable to advance knowledge on issues important to this population. We have argued that these terms can be ableist when they reduce autistic people to perceived deficits, and when they are used without examining how social contexts contribute to disability (3, 20, 21). Important to this logic are theoretical commitments from sociolinguistic research traditions showing that *no* terms or discourses express value-neutral facts (22). Instead, language (including scientific language) is imbued with ideological and ethical dimensions.

Here, we extend our previous arguments by asserting that research underpinned by unexamined ableist ideologies has no claim to scientific accuracy. Instead, ableism is historically intertwined with research programs that were eventually debunked, or are now understood to have much less explanatory power than initially proposed. We explore three such programs, including: (a) etiology, identification, and prevalence; (b) descriptions and theoretical explanations, and (c) interventions.

## False leads

### Autism etiology, identification, and prevalence

#### Psychogenesis

Delineating causal mechanisms of autism and how they contribute to diagnostic prevalence have been top research and funding priorities (18). Psychogenic theories emerged early in the history of autism research, and stemmed from Freudian theories of psychosexual development. One version purported that mothers’ rejection of their children resulted in insufficient parent–child bonding and caused their children to become autistic. Bettelheim’s (23) iteration of this theory is most well-known, but Kanner (24) implied similar sentiments regarding the parents of autistic children (focusing most often on mothers) in his original case report. He later expounded a causal link between parents’ behavior and their children’s autism (25), writing how the “[m]aternal lack of genuine warmth is often conspicuous in the first visit to the clinic” (p. 422), and that children were “kept neatly in refrigerators which did not defrost” (p. 425).

Both Kanner and Bettelheim relied on ableists and misogynistic assumptions to connect mothers’ behavior (their education, participation in work, and perceived warmth) to their autistic children’s perceived aloofness. With more rigorous investigation, the notion that mothers’ affect made their children autistic has since been widely rejected. For example, research has shown that autistic children are as securely attached to their mothers as typically developing children (26). To reflect updated research, a recently created and validated measure of autism knowledge regards autistic children showing affection, attachments, and empathy as facts (27). Essentially, the evidence for these ideas remained largely speculative and based exclusively on unsystematic and ableist clinical impressions of parents and their children—undermining any claims to scientific accuracy. Still, the damage this work caused families is well established, including for example the removal of autistic children from their homes at Bettelheim’s recommendation (28), and further stigmatization of autistic people (29). Additionally, Douglas (30) argues that these theories have been repurposed from cause to cure, with mothers no longer being blamed for being the root of their child’s autism, but instead blamed for their lack of recovery from it.

#### Toxicity and biogenesis

Psychogenic causal theories were eventually displaced by biogenic causal theories that focused on external toxins, with Bernard Rimland’s ideas in particular gaining traction throughout the 1960s – 1990s. Rimland asserted that autism had biological origins similar to disorders like phenylketonuria, which is caused by a genetic inability to break down phenylalanine and, without treatment, leads to intellectual disabilities (31). Rimland either developed or promoted several biology-based theories, including that autism is caused by toxicity from sources such as vaccines (32), insufficient digestion of gluten and casein (33, 34), and heavy metals in the bloodstream (35). None of these theories were based on strong supporting evidence at the time Rimland proposed them, and are now widely considered debunked (36). Central to their proliferation are ableist and stigmatizing notions that autism is the result of biological “damage” that negatively impacts cognitive and social development, and that biological causes, if identified, could lead to simple to manage cures. Although these theories have been rejected by much of the scientific community, discourses about autism that invoke biological perturbations (often from external ‘toxins’) frame much of the professional and public understanding of autism, which can be a significant source of stigmatization.

#### Biomarkers

Autism is defined behaviorally and diagnosed observationally, resulting in significant variability in clinical evaluation and practice. To further standardize diagnosis and clinical trials, a major focus within autism research has been a search for quantifiable biomarkers to aid early detection and serve as targets for intervention. However, to date biomarker studies have produced inconsistent and contradictory results, leading a recent review to conclude that there is a biomarker “replication crisis” and “currently no response biomarker to inform ASD clinical trials” [(37), p. 23]. The search for autism-specific biomarkers is also complicated by its notorious heterogeneity that is not likely to be associated with a uniform underlying physiology (38).

Although biomarker research offers potential for identifying biological contributors to disabling medical conditions that disproportionately co-occur with autism (e.g., epilepsy, sleep disorders, digestive issues, hyperacusis), efforts to reduce autism itself to biology ignores the social and developmental contexts in which neurobiological differences manifest as social disabilities, and reifies deficit frameworks that presume disability is intrinsic to the individual. As these efforts continue, it remains incumbent on biomarker researchers to articulate how biomarkers will improve identification and clinical care, as biomarker testing is often more labor-intensive, more invasive, and less accessible to people in need than established methods. Further, biomarker researchers should address concerns from the autistic community about the underlying motivation for biological research funding [e.g., cure and prevent autism; (39)], which often comes at the expense of other funding and research needs prioritized by autistic people (40–46).

#### The autism epidemic

Rimland was among several researchers who made ableist claims that increasing numbers of autism diagnoses constituted an “autism epidemic” (3), and that increases in cases were due to environmental factors like toxins or vaccines. These claims lack empirical support, and systematic investigations into vaccines have not shown even correlational links to increases in autism prevalence (36). Current researcher consensus about changing prevalence estimates is that increases are due to differences in identification methods across survey years, greater awareness and improved differential diagnosis that enable appropriate identification of autistic people from minoritized backgrounds, and improved service access that incentivizes diagnosis (47–49). For example, a rise in autism diagnoses among United States children recently occurred particularly for historically underrepresented non-white children and girls (50). Researchers who continue to cleave to the notion of an autism epidemic tend not to sufficiently account for these alternative explanations. Still, claims of an autism epidemic continue to be promoted by a small group of researchers and influential public figures (51, 52).

The ableist ideologies that accompany concerns about an “autism epidemic” are readily apparent, and are mobilized in part to promote increased investment in cause/cure research. In an essay linking vaccines and autism, Rimland (32) quoted a parent of an autistic child who argued that parents would know – without autism awareness campaigns – if their child was “not talking yet and does not do anything except sit there flapping his hands” (p. 261). Rimland and the parent he quoted reduced autistic children to their non-normative behavior, and conveyed that more autistic people is cause for alarm.^1^ However, recent estimates indicate that the vaguely-defined group of autistic children referenced in the parent’s quotation do not account for a substantial portion of the increase in diagnoses (54). Researchers have also expressed alarmist, ableist concerns about the “economic burden” of autistic people [Blaxill et al. (55), in a recently retracted study]. However, recent efforts to model the cost of autistic people have been critiqued on methodological and ideological grounds because they inappropriately assume that autistic people’s economic contributions are zero, that non-autistic people are cost-free, and that perceived financial cost is an informative marker for determining autistic people’s right to exist (56). Once again, ableist assumptions about autism have undermined rigorous evaluation of available evidence on autism prevalence. These stigmatizing, ableist claims reach the consciousness of autistic people and may become internalized; as one autistic adult argued, vaccine-autism fears suggest society views having a deadly disease as worse than autism (57).

### Characterizing and theorizing autism

#### Echolalia

Kanner (25) described echolalia in autistic children, which is the repetition of the speech of others, as a “…semantically and conversationally valueless or grossly distorted memory exercise” (p. 243). In the following decades, psychoanalytic and behaviorist researchers considered autistic echolalia to be non-communicative and inwardly focused. Interventions were developed to decrease its occurrence (58) under the ableist assumption that echolalia interferes with “real” social interaction and ultimately, social development (59). These conclusions did not stem from in-depth, systematic study of the social or interactional contexts in which echolalia was produced, the impacts of echolalia on interaction, or autistic people’s input. Instead, they were based on clinical reports generated with seemingly *a priori* assumptions that the non-normative nature of echolalia was evidence enough that it was not worthwhile.

Autism researchers who studied language and communication *in context* soon took a more nuanced approach to conceptualizing echolalia, and proposed that echolalia could have communicative and interactive utility [see Gernsbacher et al. (59) and Sterponi and Shankey (60), for summaries], such as language-building imitation (61). Critical to these programs of research are theoretical commitments and methodological points of departure that seek to describe what autistic people *do* in interaction, rather than to seek out deficits by honing in on any form of conduct that appears to differ from a (usually idealized) non-autistic standard. For example, in their qualitative case study, Sterponi and Shankey (62) describe how an autistic child deployed echolalia in creative ways (e.g., by adjusting prosodic contours or adding/subtracting lexical items from the original utterance) to achieve a variety of interactional ends, such as redirection, expressing alignment, and projecting affective and epistemic stance. This research builds on prior systematic, empirical descriptions of autistic interactions showing the interactional relevance of echolalia (63–65) that sharply contrasts with previous, deficit-driven research that lacked a rigorous empirical basis.

#### Social motivation

In keeping with deficit-based descriptions, researchers have developed deficit-based theories to explain how features of autism (such as echolalia) develop and co-occur. The Social Motivation hypothesis purports that autistic people have an innately reduced interest in social interaction, and are instead inwardly focused, which stems from differences in neurobiology that affect the processing of social rewards (66). These early differences are thought to culminate in diminished participation in, and ultimately capacity for, social interaction with others. This theoretical framing has led to interventions designed to increase the “reward value” of early social interactions [for example via oxytocin administration, which has shown null or negative effects across several studies; see (21, 67)] in an effort to reroute social development to a more typical pathway (61, 68).

However, Jaswal and Akhtar (61) have pointed out three problems regarding the assumptions that underpin this theory: (a) autistic people express that they do desire interactions and relationships with others, (b) there are alternative explanations for the differences in social presentation evidenced by autistic people that do not assume reduced social motivation, and (c) social motivation is not a ‘within-person’ phenomenon; it involves one’s social conduct, others’ interpretations of their social conduct, and others’ contingent social conduct based on those interpretations (69). Indeed, empirical work has found that autistic adult’s social motivation has little predictive value for social interaction outcomes (70). Social motivation theories are one of many theoretical approaches that use deficit-based bridging assumptions to link deficit-based descriptions of autism (e.g., decreased eye-contact, differences in signaling emotion) to an explanatory framework, which can have stigmatizing effects on autistic people (10).

#### Broken mirrors

A related theoretical framework posits that autistic people lack neural activation in “mirror neuron” networks of the brain, which are purported to enable a simulated experience of others’ actions by activating neural pathways during action observation that are also activated during action production (71). According to the theory, autistic people are unable to understand the goals, intentions, or affective motivations that underlie others’ actions because the activation of these pathways are attenuated, and they do not have the simulated experience of producing an action when observing one. On this basis, autistic people’s neurology was described as “broken” and in need of fixing to restore functioning (72). This theory rose to prominence in the early 2000s and led to interventions focused on improving autistic children’s ability to imitate others, a skill thought to depend on and possibly enhance mirror neuron activation (73).

In a forum discussion of mirror neuron findings (74), Gernsbacher provides provocative counterpoints to this theory, noting that many of the findings showing decreased activation of mirror neuron networks in autistic as compared non-autistic controls have not consistently replicated. Indeed, many findings locating mirror neuron networks in non-autistic groups have not held up in meta-analytic investigations either. She also notes that the interventions that arose from mirror neuron theories have limited empirical support. Finally, Gernsbacher links the development of mirror neuron theories to explain autism to prejudicial bias (i.e., ableism). The ableism motivating this theory is especially apparent in the dehumanizing language used to articulate it, which included that autistic people lack a capacity central to human evolution.

### Autism interventions

#### Young autism project

In the United States, applied behavior analysis (ABA) is a widely-implemented form of therapy for autistic people, popularized by Lovaas’ Young Autism Project. ABA designed for autistic people was derived from similar, now disavowed, strategies used to prevent children from developing traits perceived to be inconsistent with their sex assigned at birth, and to prevent future same sex attraction (75, 76). Lovaas published a seminal study in the late 1980s claiming that autistic children who participated in intensive ABA therapy became indistinguishable from their non-autistic peers (77), but this finding has not replicated (78). Indeed, the vast majority of ABA studies do not meet basic quality standards such as randomization and masked assessment (79, 80), and those that do show only modest improvements in autistic children’s cognitive development (a finding that also has not been replicated after more than 20 years). Despite the lack of high-quality evidence, many ABA providers have advertised their services as a gold-standard, scientifically-proven cure for autism (77).

Lovaas’ ableist views about autistic people are familiar to researchers in academic traditions critical of ABA (1), but are rarely acknowledged by ABA proponents. For example, in interviews, Lovaas referred to autistic children as inhuman and promoted physical abuse–including making autistic people fear for their lives– as a means to promote behavior change (81, 82). Further, Lovaas’ focus on encouraging autistic people to suppress autistic traits such as “stimming” (e.g., hand flapping, rocking, or repetitive vocalizations) so that they appear neurotypical further stigmatizes these behaviors, despite the fact that many autistic people describe stimming as an expression of joy or a valuable coping mechanism (83).

#### Defeat autism now! protocol

Consistent with his biogenic theories of autism causation, Rimland promoted the use of various therapies that he marketed as curative via his Autism Research Institute, through which he developed the Defeat Autism Now! (DAN!) protocol. This protocol was administered by DAN! doctors trained to implement strategies to remove toxins from the body, which were thought to be introduced through external influences such as diets and vaccines. Procedures included removing heavy metals from the bloodstream (i.e., chelation therapy), gluten-and casein-free diets, vitamin therapy, hyperbaric oxygen therapy, and the avoidance of childhood vaccines. These therapies were introduced with flimsy evidence of effectiveness, and insufficient attention to potential harms^2^– but Rimland felt that the need to decrease or cure characteristics associated with autism was so pressing that procedures with even just anecdotal or hypothetical support were worthwhile (84).

Subsequently, many studies have been published that refute the efficacy of these strategies (85–87), and calls have been issued for discontinuing their use due to significant harms, including death (88). While Rimland’s approaches garnered contemporary criticism from many researchers, he gained significant traction with many medical providers and families, and is recognized as having enormous and enduring influence on the care autistic children receive [(84, 89, 90)]. An analysis of Google search data shows that these theories involving gluten and heavy metals still garnered significant public interest in 2019 (with renewed interest since the onset of the COVID-19 pandemic), and vaccines remain the topic most associated with questions relating to the cause of autism on Google (91).

Underlying the continued dissemination of these intervention programs are ableist ideologies positioning autism as such an undesirable state of affairs that: (1) any possibility to reduce its occurrence is worthwhile, regardless of a lack of evidence, and (2) there is no need to consider harms, because being autistic is worse than any potential harm (92). As a result of the poor science backing much autism intervention research, it is unclear if they have resulted in long-term positive impacts for autistic people (78, 79, 93), although it is likely that many of these programs contribute to stigmatization and trauma (94, 95).

## Discussion

In this paper, we offer a counter argument to the insinuation that researchers have a choice between rejecting ableism and striving for scientific accuracy. In fact, history shows that ableism and poor autism science have gone hand in hand. Many autism research programs that have either been abandoned or have become much less influential may have gained initial traction because the ableist assumptions underpinning them were taken as givens, even though they were not backed by rigorous evidence. These include assumptions about the etiology and prevalence of autism, descriptions and theorizations about autistic people’s social conduct, and ways to support autistic people. The assumed validity of these theories further encourages poor research practices and confirmation bias (e.g., elective reporting, p-hacking, hypothesizing after results are known, etc.). Until the field of autism research explicitly addresses the link between ableism and poor autism science, new programs of research will continue to emerge that have little to offer in terms of advancing knowledge, while also potentially causing significant harm–including stigmatization– to autistic people and their families (2).

Alternatively, recent efforts to reject ableism have led to promising empirical and theoretical advances, such as the program of research underpinned by the *Double Empathy Problem* (96–98), efforts to understand features of autism using neurodiversity frameworks (99–101), rigorous guidelines for conducting co-produced research with autistic people (102), pilot research on programs designed to reduce social stigma (103), and approaches to promote autistic flourishing that prioritize capabilities such as affiliation and health via systems change, rather than encouraging autistic people to “overcome” perceived deficits (104). Anti-ableist research on supporting autistic people in their daily lives also shows promise, such as efforts to improve quality of life measures that are specific and relevant to autistic people (105) research that can be used to rigorously test interventions that aim to improve their wellbeing. Each of these programs will need to be refined and improved over time through additional research, but illustrate the potential of anti-ableist work coinciding with scientific rigor. Ultimately, anti-ableism efforts may be a requirement for, rather than in conflict with, academic rigor.

## Author contributions

KB-B proposed the manuscript, drafted the outline, wrote the initial draft, and edited the draft. SK contributed to the conceptualization of the manuscript, added to the outline, and edited the draft. NS contributed to the conceptualization of the manuscript, added to the outline, drafted text, and edited the draft. MG contributed to the conceptualization of the manuscript, and edited the draft. HN contributed to the manuscript outline, and edited the draft. MB contributed to the conceptualization of the manuscript, drafted text, and edited the draft. All authors contributed to the article and approved the submitted version.

## Funding

Open access fees were funded by the Argyelan Family Fund and the Open Access Fund of Boston College.

## Conflict of interest

KB-B has previously received fees for consulting with school districts on intervention practices for autistic children and teaches courses on autism interventions in her role as an Associate Professor of Special Education. She has also accepted speaker fees to discuss her work on research quality, adverse events, researcher conflicts of interest as they pertain to autism intervention research, and ableist language. She also receives royalties for a co-edited book titled Clinical Guide to Early Interventions for Children with Autism, published by Springer. NS has previously received speaker fees to discuss his research investigating social cognition and interaction in autism, and to share his views on the state of autism research. SK has previously received research funds from the Autism Intervention Research Network on Physical Health, and teaches courses on neurodiversity studies and social construction of disability in his role as a university professor. MB has previously received speaker fees for discussing their research on ethics and autism research. They are also an advisor for Information Autism, and on an advisory board for an education service for Neurodivergent children, young people, and adults in Scotland.

The remaining authors declare that the research was conducted in the absence of any commercial or financial relationships that could be construed as a potential conflict of interest.

## Publisher’s note

All claims expressed in this article are solely those of the authors and do not necessarily represent those of their affiliated organizations, or those of the publisher, the editors and the reviewers. Any product that may be evaluated in this article, or claim that may be made by its manufacturer, is not guaranteed or endorsed by the publisher.

## Footnotes

^1^Ableist panic of this sort came to a crescendo in the mid 2000’s, with initiatives such as the “Ransom Notes” campaign launched by the New York University Child Study Center, where large billboard displays containing notes purporting to be from neurodevelopmental disabilities such as autism notified parents that they had stolen and harmed their children (49).

^2^In one study, Rimland reported that a drug he promoted was found to “induce adverse side effects in only about 19% of the children” [(78), p. 69], which he concluded was too low to take into consideration. He also asserted that high doses of vitamins did not have the potential for harm.

## References

[1] Botha M. Academic, activist, or advocate? Angry, entangled, and emerging: a critical reflection on autism knowledge production. Front Psychol. (2021) 12:727542. doi: 10.3389/fpsyg.2021.727542 34650484 PMC8506216

[2] Botha M Cage E. “Autism research is in crisis”: a mixed method study of researcher’s constructions of autistic people and autism research. Front Psychol. (2022) 13:7397. doi: 10.3389/fpsyg.2022.1050897 PMC9730396 36506950

[3] Bottema-Beutel K Kapp SK Lester JN Sasson NJ Hand BN. Avoiding ableist language: suggestions for autism researchers. Autism Adulthood. (2021) 3:18–29. doi: 10.1089/aut.2020.0014, PMID: 36601265 PMC8992888

[4] Lewis T.L. (2022). Working Definition of Ableism. Talila A Lewis. Available at: https://www.talilalewis.com/blog (Accessed June 11, 2023).

[5] Goffman E. Stigma: Notes on the Management of Spoiled Identity. New York, NY: Simon and Schuster. (1963).

[6] Gillespie-Lynch K Kapp SK Brooks PJ Pickens J Schwartzman B. Whose expertise is it? Evidence for autistic adults as critical autism experts. Front Psychol. (2017) 8:438. doi: 10.3389/fpsyg.2017.00438 28400742 PMC5368186

[7] Turnock A Langley K Jones CR. Understanding stigma in autism: a narrative review and theoretical model. Autism Adulthood. (2022) 4:76–91. doi: 10.1089/aut.2021.0005, PMID: 36605561 PMC8992913

[8] Gernsbacher MA. On not being human. APS observer. (2007) 20:5. PMC2709287 19603081

[9] Gernsbacher MA Dawson M Mottron L. Autism: common, heritable, but not harmful. Behav Brain Sci. (2006) 29:413–4. doi: 10.1017/S0140525X06319097, PMID: 25506106 PMC4263218

[10] Kapp S. How social deficit models exacerbate the medical model: autism as case in point. Autism Policy Pract. (2019) 2:3–28.

[11] Rozenkrantz L D’Mello AM Gabrieli JD. Enhanced rationality in autism spectrum disorder. Trends Cogn Sci. (2021) 25:685–96. doi: 10.1016/j.tics.2021.05.004, PMID: 34226128

[12] Cowen T. Autism as academic paradigm. The Chronicles of Higher Education (2009). Available at: https://www.chronicle.com/article/autism-as-academic-paradigm (Accessed August 29, 2023).

[13] Gernsbacher MA. On not being human. Observer. (2007) 20:5–32. PMC4266404 25520547

[14] Sinclair J. Don't mourn for us. Autonomy Crit J Interdisc Autism Stud. (2012) 1

[15] Russell G Kapp SK Elliott D Elphick C Gwernan-Jones R Owens C. Mapping the autistic advantage from the accounts of adults diagnosed with autism: a qualitative study. Autism Adulthood. (2019) 1:124–33. doi: 10.1089/aut.2018.0035, PMID: 31058260 PMC6493410

[16] Urbanowicz A Nicolaidis C Houting JD Shore SM Gaudion K Girdler S. An expert discussion on strengths-based approaches in autism. Autism Adulthood. (2019) 1:82–9. doi: 10.1089/aut.2019.29002.aju, PMID: 36601531 PMC8992818

[17] Gernsbacher MA. The true meaning of research participation. Observer. (2007) 20:5–13. PMC4260421 25505828

[18] Singer A Lutz A Escher J Halladay A. A full semantic toolbox is essential for autism research and practice to thrive. Autism Research. (2023) 16:497–501., PMID: 36508163 10.1002/aur.2876

[19] Tan DW. Early-career autism researchers are shifting their research directions: tragedy or opportunity? Autism Adulthood. (2023). doi: 10.1089/aut.2023.0021 [Epub ahead of print]. PMC10468548 37663448

[20] Natri HM Abubakare O Asasumasu K Basargekar A Beaud F Botha M. Anti-ableist language is fully compatible with high-quality autism research: response to Singer et al. (2023). Autism Res. (2023) 16:673–6. doi: 10.1002/aur.2928, PMID: 37087601

[21] Kapp SK Damian M Sara R. Models of helping and coping with autism In: The Routledge International Handbook of Critical Autism Studies. Eds. Milton D. Ryan S. Abingdon, Oxon: Routledge. (2022) 255–69.

[22] Bloemmaert J. Discourse: A Critical Introduction. Cambridge, UK: Cambridge University Press. (2005).

[23] Harris L Gilmore D Longo A Hand BN. Patterns of US federal autism research funding during 2017–2019. Autism. (2021) 25:2135–9. doi: 10.1177/13623613211003430, PMID: 33765838

[24] Bettelheim B. The Empty Fortress: Infantile Autism and the Birth of the Self. New York, NY: Simon and Schuster. (1967).

[25] Kanner L. Autistic disturbances of affective contact. Nervous Child. (1943) 2:217–50. 4880460

[26] Kanner L. Problems of nosology and psychodynamics of early infantile autism. Am J Orthopsychiatry. (1949) 19:416–26. doi: 10.1111/j.1939-0025.1949.tb05441.x, PMID: 18146742

[27] Gernsbacher MA Dawson M Hill Goldsmith H. Three reasons not to believe in an autism epidemic. Curr Dir Psychol Sci. (2005) 14:55–8. doi: 10.1111/j.0963-7214.2005.00334.x, PMID: 25404790 PMC4232964

[28] Gillespie-Lynch K Brooks PJ Someki F Obeid R Shane-Simpson C Kapp SK. Changing college students’ conceptions of autism: an online training to increase knowledge and decrease stigma. J Autism Dev Disord. (2015) 45:2553–66. doi: 10.1007/s10803-015-2422-9, PMID: 25796194

[29] Crowell JA Keluskar J Gorecki A. Parenting behavior and the development of children with autism spectrum disorder. Compr Psychiatry. (2019) 90:21–9. doi: 10.1016/j.comppsych.2018.11.007 30658339

[30] Douglas P. Refrigerator mothers. J Mother Initiat Res Commun Involv. (2014) 5:94–114.

[31] Rimland B. Infantile autism: status of research. Can Psychiatr Assoc J. (1974) 19:130–3. doi: 10.1177/070674377401900203 4829412

[32] Rimland B. The autism epidemic, vaccinations, and mercury. J Nutr Environ Med. (2000) 10:261–6. doi: 10.1080/13590840020013248

[33] Lewis L. Special Diets for Special Kids: Understanding and Implementing a Gluten and Casein Free Diet to Aid in the Treatment of Autism and Related Disorders. Arlington, TX: Future Horizons (1998).

[34] Reichelt KL Ekrem J Scott H. Gluten, milk proteins and autism: dietary intervention effects on behavior and peptide secretion. J Appl Nutr. (1990) 42:1–11.

[35] Rimland B Larson GE. Hair mineral analysis and behavior: an analysis of 51 studies. J Learn Disabil. (1983) 16:279–85. doi: 10.1177/002221948301600507 6348191

[36] Taylor LE Swerdfeger AL Eslick GD. Vaccines are not associated with autism: an evidence-based meta-analysis of case-control and cohort studies. Vaccine. (2014) 32:3623–9. doi: 10.1016/j.vaccine.2014.04.085 24814559

[37] Parellada M Andreu-Bernabeu Á Burdeus M San José Cáceres A Urbiola E Carpenter LL. In search of biomarkers to guide interventions in autism spectrum disorder: a systematic review. Am J Psychiatr. (2023) 180:23–40. doi: 10.1176/appi.ajp.21100992, PMID: 36475375 PMC10123775

[38] Molloy CJ Gallagher L. Can stratification biomarkers address the heterogeneity of autism spectrum disorder? Ir J Psychol Med. (2022) 39:305–11. doi: 10.1017/ipm.2021.73, PMID: 34823622

[39] Pukki H Bettin J Outlaw AG Hennessy J Brook K Dekker M. Autistic perspectives on the future of clinical autism research. Autism Adulthood. (2022) 4:93–101. doi: 10.1089/aut.2022.0017, PMID: 36601072 PMC9242721

[40] Cage E Crompton CJ Dantas S Strachan K Birch R Robinson M. What are the autism research priorities of autistic adults in Scotland? Psy ArXiv. (2022). doi: 10.31234/osf.io/nbq8h [Preprint]. PMC11401337 38311602

[41] Dey I Chakrabarty S Nandi R Shekhar R Singhi S Nayar S. Autism community priorities in diverse low-resource settings: a country-wide scoping exercise in India. Autism. (2023):136236132311540. doi: 10.1177/13623613231154067 [Epub ahead of print]. PMC10771024 36999343

[42] Fletcher-Watson S Apicella F Auyeung B Beranova S Bonnet-Brilhault F Canal-Bedia R. Attitudes of the autism community to early autism research. Autism. (2017) 21:61–74. doi: 10.1177/1362361315626577 26975669

[43] Frazier TW Dawson G Murray D Shih A Sachs JS Geiger A. Brief report: a survey of autism research priorities across a diverse community of stakeholders. J Autism Dev Disord. (2018) 48:3965–71. doi: 10.1007/s10803-018-3642-6 Accessed August 29, 2023., PMID: 29948533

[44] Pellicano E Dinsmore A Charman T. What should autism research focus upon? Community views and priorities from the United Kingdom. Autism. (2014) 18:756–70. doi: 10.1177/1362361314529627, PMID: 24789871 PMC4230972

[45] Putnam OC Eddy G Goldblum J Swisher M Harrop C. How autistic adults’ priorities for autism research differ by gender identity: a mixed-methods study. Womens Health. (2023) 19:174550572311603. doi: 10.1177/17455057231160342 PMC10071159 36999307

[46] Warner G Parr JR Cusack J. Workshop report: establishing priority research areas to improve the physical health and well-being of autistic adults and older people. Autism Adulthood. (2019) 1:20–6. doi: 10.1089/aut.2018.0003

[47] Gernsbacher MA Dissanayake C Goldsmith HH Mundy PC Rogers SJ Sigman M. Autism and deficits in attachment behavior. Science. (2005) 307:1201–3. doi: 10.1126/science.307.5713.1201, PMID: 15731426 PMC4301420

[48] Harris E. Autism prevalence has been on the rise in the US for decades– and that’s progress. JAMA. (2023) 329:1724–6. doi: 10.1001/jama.2023.6078, PMID: 37133882

[49] Mandell D Lecavalier L. Should we believe the Centers for Disease Control and Prevention’s autism spectrum disorder prevalence estimates? Autism. (2014) 18:482–4. doi: 10.1177/1362361314538131, PMID: 26005737

[50] Maenner MJ Warren Z Williams AR Amoakohene E Bakian AV Bilder DA. Prevalence and characteristics of autism spectrum disorder among children aged 8 years—autism and developmental disabilities monitoring network, 11 sites, United States, 2020. MMWR Surveill Summ. (2023) 72:1–14. doi: 10.15585/mmwr.ss7202a1, PMID: 36952288 PMC10042614

[51] Dufault R Lukiw WJ Crider R Schnoll R Wallinga D Hall-Lande J. A macroepigenetic approach to identify factors responsible for the autism epidemic in the United States. Clinical Epigenetics. (2012) 4:1–12. 22490277 10.1186/1868-7083-4-6 PMC3378453

[52] Miller M. E. (2015). The GOP’s Dangerous ‘Debate’ on Vaccines and Autism Wash Post. Available at: https://www.washingtonpost.com/news/morning-mix/wp/2015/09/17/the-gops-dangerous-debate-on-vaccines-and-autism/ (Accessed August 29, 2023).

[53] Kras JF. The “ransom notes” affair: when the neurodiversity movement came of age. Disabil Stud Q. (2010) 30:1065. doi: 10.18061/dsq.v30i1.1065

[54] Hughes MM Shaw KA DiRienzo M Durkin MS Esler A Deth R. The prevalence and characteristics of children with profound autism, 15 sites, United States, 2000-2016. Public Health Rep. (2023):333549231163551. doi: 10.1177/00333549231163551 [Epub ahead of print]. 37074176 PMC10576490

[55] Blaxill M Rogers T Nevison C. Autism tsunami: the impact of rising prevalence on the societal cost of autism in the United States. J Autism Dev Disord. (2022) 52:2627–43. doi: 10.1007/s10803-021-05120-7, PMID: 34278527 PMC9114071

[56] Luterman S. (2021). Contentious Study Prompts Backlash from Autism Researchers. Available at https://www.spectrumnews.org/news/contentious-study-prompts-backlash-from-autism-researchers/ (Accessed May 30, 2023).

[57] Botha M Dibb B Frost DM. " autism is me": an investigation of how autistic individuals make sense of autism and stigma. Disabil Soc. (2022) 37:427–53. doi: 10.1080/09687599.2020.1822782

[58] Neely L Gerow S Rispoli M Lang R Pullen N. Treatment of echolalia in individuals with autism spectrum disorder: a systematic review. Rev J Autism Dev Disord. (2016) 3:82–91. doi: 10.1007/s40489-015-0067-4

[59] Gernsbacher MA Morson EM Grace EJ. Language and speech in autism. Ann Rev Linguist. (2016) 2:413–25. doi: 10.1146/annurev-linguistics-030514-124824, PMID: 28127576 PMC5260808

[60] Sterponi L Shankey J. Rethinking echolalia: repetition as interactional resource in the communication of a child with autism. J Child Lang. (2014) 41:275–304. doi: 10.1017/S0305000912000682, PMID: 23469804

[61] Jaswal VK Akhtar M. Being versus appearing socially uninterested: challenging assumptions about social motivation in autism. Behav Brain Sci. (2019) 42:1–73. doi: 10.1017/S0140525X18001826 29914590

[62] Local J Wootton A. Interactional and phonetics aspects of immediate echolalia in autism: a case study. Clin Linguist Phon. (1995) 9:155–84. doi: 10.3109/02699209508985330

[63] Tarplee C Barrow E. Delayed echoing as an interactional resource: a case study of a 3-year-old child on the autistic spectrum. Clin Linguist Phonet. (1999) 6:449–82.

[64] Wootton A. An investigation of delayed echoing in a child with autism. Language. (1999) 19:359–81.

[65] Clements CC Zoltowski AR Yankowitz LD Yerys BE Schultz RT Herrington JD. Evaluation of the social motivation hypothesis of autism: a systematic review and meta-analysis. JAMA Psychiat. (2018) 75:797–808. doi: 10.1001/jamapsychiatry.2018.1100, PMID: 29898209 PMC6143096

[66] Moerkerke M Daniels N Van der Donck S Tibermont L Tang T Debbaut E. Can repeated intranasal oxytocin administration affect reduced neural sensitivity towards expressive faces in autism? a randomized controlled trial. J Child Psychol Psychiatry Adv. (2023). doi: 10.1111/jcpp.13850 [Epub ahead of print]. 37278339

[67] Bradshaw J Koegel LK Koegel RL. Improving functional language and social motivation with a parent-mediated intervention for toddlers with autism spectrum disorder. J Autism Dev Disord. (2017) 47:2443–58. doi: 10.1007/s10803-017-3155-8, PMID: 28536956

[68] Gernsbacher MA. Toward a behavior of reciprocity. J Dev Process. (2006) 1:139–52. PMID: 25598865 PMC4296736

[69] Morrison KE DeBrabander KM Jones DR Ackerman RA Sasson NJ. Social cognition, social skill, and social motivation minimally predict social interaction outcomes for autistic and non-autistic adults. Front Psychol. (2020) 11:3282. doi: 10.3389/fpsyg.2020.591100 PMC7723837 33324295

[70] Rizzolatti G Fabbri-Destro M. Mirror neurons: from discovery to autism. Exp Brain Res. (2010) 200:223–37. doi: 10.1007/s00221-009-2002-3, PMID: 19760408

[71] Ramachandran VS Oberman LM. Broken mirrors. Sci Am. (2006) 295:62–9. doi: 10.1038/scientificamerican1106-62, PMID: 17076085

[72] Iacoboni M Mazziotta JC. Mirror neuron system: basic findings and clinical applications. Ann Neurol. (2007) 62:213–8. doi: 10.1002/ana.21198, PMID: 17721988

[73] Gallese V Gernsbacher MA Heyes C Hickok G Iacoboni M. Mirror neuron forum. Perspect Psychol Sci. (2011) 6:369–407. doi: 10.1177/1745691611413392, PMID: 25520744 PMC4266473

[74] Conine DE Campau SC Petronelli AK. LGBTQ+ conversion therapy and applied behavior analysis: a call to action. J Appl Behav Anal. (2022) 55:6–18. doi: 10.1002/jaba.876, PMID: 34407211

[75] Dawson M. The Misbehaviour of Behaviourists: Ethical Challenges to the Autism-ABA Industry Autism Crisis; (2004). Available at: https://www.sentex.ca/~nexus23/naa_aba.html (Accessed May 30, 2023).

[76] Lovaas OI. Behavioral treatment and normal educational and intellectual functioning in young autistic children. Journal of Consulting and Clinical Psychology. (1987) 55:3. 3571656 10.1037//0022-006x.55.1.3

[77] Gernsbacher MA. Is one style of early behavioral treatment for autism 'Scientifically proven?'. J Dev Process. (2003) 7:19–26. PMC4266398 25520761

[78] Bottema-Beutel K LaPoint SC Kim SY Mohiuddin S Yu Q McKinnon R. An evaluation of intervention research for transition-age autistic youth. Autism. (2023) 27:890–904. doi: 10.1177/13623613221128761, PMID: 36189778

[79] Sandbank M Bottema-Beutel K Crowley S Cassidy M Dunham K Feldman JI. Project AIM: autism intervention meta-analysis for studies of young children. Psychol Bull. (2020) 146:1–29. doi: 10.1037/bul0000215, PMID: 31763860 PMC8783568

[80] Chance P. A conversation with Ivar Lovaas. Psychol Today. (1974) 7:76–84.

[81] Grant A. Screams, slaps, and love. Life. (1965):87–97.

[82] Kapp SK Steward R Crane L Elliott D Elphick C Pellicano E. ‘People should be allowed to do what they like’: autistic adults’ views and experiences of stimming. Autism. (2019) 23:1782–92. doi: 10.1177/1362361319829628, PMID: 30818970 PMC6728747

[83] Rimland B. Controversies in the treatment of autistic children: vitamin and drug therapy. J Child Neurol. (1988) 3:S68–72. doi: 10.1177/0883073888003001S13, PMID: 3058789

[84] Shattock P. Bernard Rimland: Parent and practitioner revolutionising the treatment of autism. The Guardian (2006). Available at: https://www.theguardian.com/news/2006/dec/06/guardianobituaries.mainsection (Accessed August 29, 2023).

[85] James S Stevenson SW Silove N Williams K. Chelation for autism spectrum disorder (ASD). Cochrane Database Syst Rev. (2015) 5:CD010766. doi: 10.1002/14651858.CD010766.pub2, PMID: 26114777 PMC6457964

[86] Mulloy A Lang R O’Reilly M Sigafoos J Lancioni G Rispoli M. Gluten-free and casein-free diets in the treatment of autism spectrum disorders: a systematic review. Res Autism Spectr Disord. (2010) 4:328–39. doi: 10.1016/j.rasd.2009.10.008

[87] Nye C Brice A. Combined vitamin B6-magnesium treatment in autism spectrum disorder. Cochrane Database Syst Rev. (2005) 4:CD003497. doi: 10.1002/14651858.CD003497.pub2 PMC7003675 16235322

[88] Sinha Y Silove N Williams K. Chelation therapy and autism. BMJ. (2006) 333:756. doi: 10.1136/bmj.333.7571.756, PMID: 17023484 PMC1592402

[89] Green VA Pituch KA Itchon J Choi A O’Reilly M Sigafoos J. Internet survey of treatments used by parents of children with autism. Res Dev Disabil. (2007) 27:70–84. doi: 10.1016/j.ridd.2004.12.002 15919178

[90] Jonkman KM Back E Staal WG Benard L van der Doelen DM Begeer S. Alternative treatments for autism: prevalence and predictors. Res Autism Spectr Disord. (2022) 98:102046. doi: 10.1016/j.rasd.2022.102046

[91] Saposnik FE Huber JF. Trends in web searches about the causes and treatments of autism over the past 15 years: exploratory Infodemiology study. JMIR Pediatr Parent. (2020) 3:e20913. doi: 10.2196/20913, PMID: 33284128 PMC7752533

[92] Dawson M Fletcher-Watson S. When autism researchers disregard harms: a commentary. Autism. (2022) 26:564–6. doi: 10.1177/13623613211031403, PMID: 34291651 PMC8814944

[93] Lord C Charman T Havdahl A Carbone P Anagnostou E Boyd B. The lancet commission on the future of care and clinical research in autism. Lancet. (2022) 399:271–334. doi: 10.1016/S0140-6736(21)01541-5, PMID: 34883054

[94] Anderson LK. Autistic experiences of applied behavior analysis. Autism. (2023) 27:737–50. doi: 10.1177/13623613221118216, PMID: 35999706

[95] McGill O Robinson A. “Recalling hidden harms”: autistic experiences of childhood applied behavioural analysis (ABA). Adv Autism. (2020) 7:269–82. doi: 10.1108/AIA-04-2020-0025

[96] Crompton CJ Ropar D Evans-Williams CV Flynn EG Fletcher-Watson S. Autistic peer-to-peer information transfer is highly effective. Autism. (2020) 24:1704–12. doi: 10.1177/1362361320919286, PMID: 32431157 PMC7545656

[97] Milton DE. On the ontological status of autism: the ‘double empathy problem’. Disabil Soc. (2012) 27:883–7. doi: 10.1080/09687599.2012.710008

[98] Sasson NJ Faso DJ Nugent J Lovell S Kennedy DP Grossman RB. Neurotypical peers are less willing to interact with those with autism based on thin slice judgments. Sci Rep. (2017) 7:1–10. doi: 10.1038/srep40700 28145411 PMC5286449

[99] Gernsbacher MA. Diverse brains. Gen Psychol. (2015) 49:29–37. 28090598 PMC5226658

[100] Kapp SK. Empathizing with sensory and movement differences: moving toward sensitive understanding of autism. Front Integr Neurosci. (2013) 7:38. doi: 10.3389/fnint.2013.00038 23745107 PMC3662878

[101] Ratto AB Bascom J da Vanport S Strang JF Anthony LG Verbalis A. Centering the inner experience of autism: development of the self-assessment of autistic traits. Autism Adulthood. (2023) 5:93–105. doi: 10.1089/aut.2021.0099, PMID: 36941856 PMC10024271

[102] Nicolaidis C Raymaker D Kapp SK Baggs A Ashkenazy E McDonald K. The AASPIRE practice-based guidelines for the inclusion of autistic adults in research as co-researchers and study participants. Autism. (2019) 23:2007–19. doi: 10.1177/1362361319830523, PMID: 30939892 PMC6776684

[103] Gillespie-Lynch K Bisson JB Saade S Obeid R Kofner B Harrison AJ. If you want to develop an effective autism training, ask autistic students to help you. Autism. (2022) 26:1082–94. doi: 10.1177/13623613211041006, PMID: 34472359

[104] Pellicano E Fatima U Hall G Heyworth M Lawson W Lilley R. A capabilities approach to understanding and supporting autistic adulthood. Nat Rev Psychol. (2022) 1:624–39. doi: 10.1038/s44159-022-00099-z, PMID: 36090460 PMC9443657

[105] Williams ZJ Cascio CJ Woynaroski TG. Measuring subjective quality of life in autistic adults with the PROMIS global–10: psychometric study and development of an autism-specific scoring method. Autism. (2023) 27:145–57. doi: 10.1177/13623613221085364, PMID: 35403453 PMC9550880

